# Diagnosis of Genital Tuberculosis in Infertile Women by Using the Composite Reference Standard

**DOI:** 10.1155/2022/8078639

**Published:** 2022-08-16

**Authors:** Riden Saxena, Kriti Shrinet, Sachchida Nand Rai, Kamal Singh, Shivi Jain, Shuchi Jain, Deeksha Singh, Shampa Anupurba, Madhu Jain

**Affiliations:** ^1^Department of Obstetrics & Gynecology, Institute of Medical Sciences, Banaras Hindu University, 221005, Varanasi, India; ^2^School of Biotechnology, Banaras Hindu University, 221005, Varanasi, India; ^3^School of Biotechnology, IFTM University, 244102, Moradabad, India; ^4^Centre of Biotechnology, University of Allahabad, 211002, Prayagraj, India; ^5^Department of Microbiology, Institute of Medical Sciences, Banaras Hindu University, 221005, Varanasi, India; ^6^Virus Research & Diagnostic Laboratory, Department of Virology, Rajendra Memorial Research Institute of Medical Science, 800007, Patna, Bihar, India; ^7^Department of Radiology, Institute of Medical Sciences, Banaras Hindu University, 221005, Varanasi, India

## Abstract

Female genital tuberculosis (FGTB) can be asymptomatic or even masquerade as other gynecological conditions. Conventional methods of FGTB diagnosis include various imaging, bacteriological, molecular, and pathological techniques that are only positive in a small percentage of patients, leaving many cases with undiagnosed condition. In the absence of a perfect diagnostic method, composite reference standards (CRSs) have been advocated in this diagnostic study. This study assesses the agreement between traditional diagnostic modalities using CRS and prevalent TB groups among different fallopian tube infertility manifestations. A total of 86 women with primary and secondary infertility were included in the study and subjected to bacteriological, pathological, and radiological examination for the diagnosis of FGTB. Results were evaluated statistically for concordance of the diagnostic tests to the CRS by sensitivity and specificity, while PPV and NPV were calculated for the performance of diagnostic tests of FGTB. We observed that 11.2% of women were found to be true positives by means of CRS. The positive findings by CRS were as follows: ultrasonography (13.9%), laparoscopy (14%), hysteroscopy (12%), GeneXpert (4.8%), culture (4.8%), polymerase chain reaction (4.8%), and histopathology (6.4%). GeneXpert and culture were found to have a perfect agreement with CRS. Hysterosalpingography, laparoscopy, and hysteroscopy have a fair agreement with CRS. Out of 43 women with tubal factor infertility, 6 women were found in the definitive TB group with mixed conditions of tubal manifestations. This study evaluates and demonstrates the reliability of the collective assessment of various diagnostic methods with CRS findings that help in identifying different TB groups of genital tuberculosis patients from all infertile patients by applying the criteria of CRS.

## 1. Introduction

FGTB is still a serious concern in low-income nations, causing substantial morbidity, particularly infertility at reproductive age [1]. Due to underreporting of cases, asymptomatic incidences, ambiguous symptomatology, and lack of effective diagnostics with high sensitivity, the exact prevalence of FGTB remains unknown [2, 3]. The reported incidence varies by country: 1% in US infertility clinics [4], 1% in Scandinavian countries [5], and 4–8% in Pakistan [6]. South Africa's share is 1% [7]. In different parts of India, the rates range from 16.1% to 19% [8]. The prevalence of female genital TB recently reported in India ranges from 45.1 cases per 100,000 women in the community-based research in the Andaman Islands to 48.5 percent among infertile women in north India [9, 10]. The pathophysiology of FGTB in infertile female patients may be considerably influenced by genetics, immunology, environment, and infection [11]. FGTB usually occurs secondary to pulmonary TB [12]. It causes a variety of nonspecific symptoms in women, ranging from infertility to irregular menstruation and pelvic pain [13]. FGTB is regarded as a chronic and asymptomatic or low symptomatic disease, so it may be difficult to diagnose in women with infertility [14]. Due to its paucibacillary nature, the diagnostic dilemma of FGTB continues to be a challenge [15]. A timely diagnosis and effective treatment may prevent it. Bacterial cultures and PCR-based diagnostics are two instances of the more cutting-edge and effective diagnostic methods that are increasingly accessible for the detection of tuberculosis [16]. The sensitivity and specificity are significantly influenced by technical factors as well, including the use of appropriate controls, standard strains, adequate conditions, and the retesting of samples with suspicious positive results [17]. The present study is assessing the combined diagnostic modality for early detection of genital tuberculosis with accuracy. According to the TB guidelines, the diagnosis of FGTB should be made based on any one of the laparoscopic features typical for FGTB, any gynecological specimen positive for acid-fast smear, or positive for mycobacterium tuberculosis (MTB) on culture, any findings consistent with FGTB on histopathology [18]. In the CRS, we included culture, GeneXpert, PCR, histology, radiography, imaging, and history for the diagnosis of FGTB. TB will be confirmed if there are two of the following AFB microscopy/histopathology/consistent feature on USG and HSG/laparoscopic or hysteroscopy features typical for FGTB or GeneXpert or culture positive in individuals with suspicion of FGTB. PCR or history with imaging features of FGTB will be considered in probable TB category, whereas only imaging features suggestive to FGTB will be grouped into possible TB group and patient tested negative for all tests will be considered as non-TB case. The primary objective of this prospective diagnostic accuracy study was to diagnose FGTB and assess TB in women with tubal factor infertility by means of CRS. We also evaluated the concordance of the diagnostic tests with respect to the CRS by sensitivity and specificity, while positive predictive value (PPV) and negative predictive value (NPV) were calculated for the performance of diagnostic tests of FGTB.

## 2. Materials and Methods

### 2.1. Study Design and Specimens

This study was conducted in the Department of Obstetrics and Gynecology and Department of Microbiology, Institute of Medical Science, Banaras Hindu University, India. The study was approved by the Ethics Committee [Dean/2018/EC/481]. During one year (December 2018 to December 2019) of research, a total of 86 women were included in the study with written consent. The unexplained and asymptomatic or low symptomatic infertility and general investigation for infertility helped to make suspicion for secondary tuberculosis. Post hoc sample size for two proportions was calculated. Only 62 women with primary (72%) and secondary (28%) infertility were selected and enrolled according to their clinical presentation. Exclusion criteria are as follows: women over the age of 45, with symptoms suggestive of pulmonary tuberculosis other than infertility, who had taken or were on a regimen of antituberculosis drugs, severe psychiatric dysfunctions, sexual disorders, infertility due to abnormality in ovulation, endocrine problems, pulmonary infections, multiple sclerosis or other autoimmune disorders, human immunodeficiency virus (HIV) and coinfections, diabetes, malnutrition, and other medical disorders like hypertension and peritoneal adhesions due to previous abdominal surgery. Control samples: 62 ETBs samples were selected from fertile women coming for medical termination of pregnancy in the family planning department.

### 2.2. Imaging Data Collection

All of the patients were subjected to a thorough clinical imaging examination such as USG, HSG, hysteroscopy, and laparoscopy. HSG was not done on a regular basis, but the results were recorded whenever it was done from the outside. When feasible, diagnostic video laparoscopy and visual hysteroscopy were performed.

#### 2.2.1. Ultrasonography (USG)

All 62 women were investigated for the presence of loculated ascites, bilateral, predominantly solid adnexal masses with scattered small calcification, thickened peritoneum, thickened omentum, and endometrial involvement on high resolution abdominal and transvaginal USG alerted to the possibility of genital tract TB [19].

### 2.3. Hysterosalpingography (HSG)

Out of 62 women, 43 women had HSG findings of tubal factor infertility. TB manifested in various forms in HSG and nonspecific changes like tubal occlusion, tubal dilatation, diverticular outpouching (salpingitis isthmic nodosa), irregular contour, and hydrosalpinx to specific patterns like pipestem tube, cotton wool plug, cobblestone tube, golf club tube, leopard skin tube, and beaded tube. In the presence of synechiae, tubal blockage in the transition zone between the isthmus and the ampulla, calcified lymph nodes, multiple constrictions, and adnexal calcifications that are irregular, linear, or nodular, TB will be highly suspected. Special features such as collar-stud abscess, T-shaped uterus, and pseudounicornuate uterus, as well as nonspecific features such as synechiae formation, uterine contour distortion, obliteration of the uterine cavity, and venous and lymphatic intravasations, may be seen as a consequence of tuberculosis. Tubal manifestations were categorized into definitive TB, probable TB, possible TB, and non-TB groups [20].

#### 2.3.1. Endoscopy

**Figure 1:**
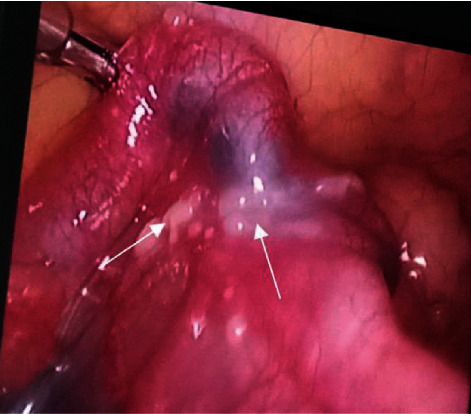
Representative laparoscopic view and arrows showing caseous nodules in FGTB case.

Out of 62 women, 50 women were undergone laparoscopy and hysteroscopy. For the diagnosis of FGTB, microcaseations, and micropolyps, fibrosed ostia, synechia bands, narrow cavity/T-shape cavity, and Asherman's syndrome are considered as diagnostic classification by hysteroscopy. During hysteroscopy, the color of the endometrium, the opening of the endometrial glands, and any TB features such as tubercles, shaggy regions, and intrauterine adhesions were all thoroughly examined. The entire pelvic and abdominal cavity, including the fallopian tubes, uterus, ovaries, Douglas pouch, uterovesical pouch, liver, peritoneum, intestines, and gall bladder, were thoroughly examined for any tuberculous lesions such as tubercles, shaggy areas, pyosalpinx, hydrosalpinx, beading of tubes, pelvic, abdominal or perihepatic adhesions, ovarian tuberculosis, tube patency, and all other abnormalities carefully studied by laparoscopy. Sacculated tubes, convoluted, fluid-filled vesicles, yellow discoloration of mesosalpinx, hydrosalpinx, lead pipe appearance, encysted fluid collection, tubo-ovarian mass, pyosalpinx, various grades of pelvic adhesions, and miliary tubercles appearances are considered as diagnostic classification by laparoscopy (Figure 1) [21].

### 2.4. Processing of Endometrial Tissue Biopsy

Endometrial biopsies (EMBs) from all 62 women were aspirated using Karman cannula no. 4, between the 20th and 25th day of menstruation in the mini operation theatre of the hospital. In the BSL-3 laboratory, each EMB sample was centrifuged for 20 minutes at 6,000 rpm in a tube containing sterile normal saline, and the pellet (about 1 mL) was transferred to a 1.5-mL Eppendorf tube containing fine glass beads up to one-third of the Eppendorf tube's capacity. For 1 minute, the material was homogenized in a tissue lyser (Bertin Technologies Pvt. Ltd) [22]. Each homogenized tissue sample was divided into three parts: GeneXpert, PCR, and culture. The colony grew on Lowenstein Jensen (L-J) media were again subjected to AFB staining and PCR.

### 2.5. GeneXpert MTB/RIF Assay

One ml of homogenized EMB was mixed with 2.0 ml of GeneXpert sample reagent. For 30 seconds, the mixture was vortexed. After allowing the sample to stand for 15 minutes at room temperature, 2 ml of the mixed sample was transferred to the test cartridge. The cartridge was inserted into the GeneXpert instrument (Cepheid). Within 2 hours, the results were reported as affirmative or negative, as well as sensitivity to the rifampicin (RIF) resistance determining region of the *rpoB* gene using molecular beacons [23].

### 2.6. DNA Extraction

DNA isolation was carried out from the homogenized EMB using the cetyl trimethylammonium bromide chloroform (CTAB-chloroform) method with slight modifications in BSL-3 Lab [24]. Thermo Scientific NanoDrop 2000 was used to analyze the quality and quantity of isolated DNA.

### 2.7. Polymerase Chain Reaction

We chose a highly conserved and restricted region of the *MPT64* (771 bp) gene encoded by the regions of difference_2_ (RD_2_). Forward and reverse sequences (5′-3′) of the primers were *ACCGAACACTCATTTCCGC* and *CTACTCCCGGAGGAATTTCG,* respectively. Reaction conditions were as follows: initial denaturation at 95°C for 5 min, 30 cycles of 95°C for 30 s, 59°C for 45 s, 72°C for 45 s, and 30 cycles of 95°C for 30 s, 59°C for 45 s, 72°C for 45 s, and 10-minute final elongation phase at 72°C [25].

### 2.8. Solid Culture

About 100 *μ*l homogenized EMB was inoculated on the L-J medium slant in a bottle and left on a horizontal plain until the inocula were absorbed. The culture bottles were incubated at 37°C. The inoculated bottles were inspected after 24 hours, 48 hours, and then once a week for the next eight weeks. AFB staining was performed from a colony grown on L-J media [26].

### 2.9. Histopathological Examination

**Figure 2:**
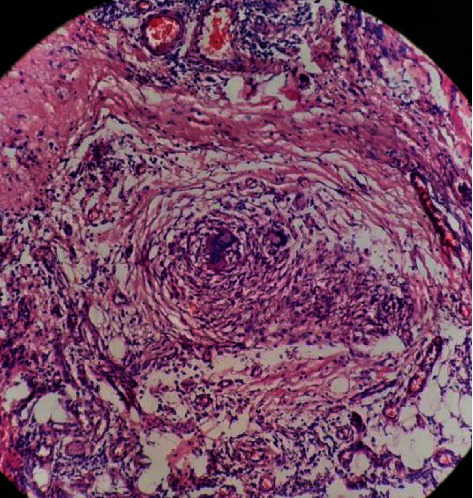
Representative granulomatous inflammation on histopathology showing well-formed granuloma with giant cell at center surrounded by epitheloid cells and lymphocytes, outermost surrounded by fibroblasts.

The biopsy specimens were cut into paraffin-embedded tissue slices and fixed in 10% formalin, hematoxylin, and eosin stains and were used to stain the sections. Caseating granuloma is indicated in samples for the diagnosis of genital tract TB, along with epithelioid cells, giant cells, fibrosis, and lymphocyte proliferation coupled with caseous necrosis (Figure 2) [22].

### 2.10. Statistical Analysis

Sensitivity, specificity, PPV, and NPV were determined by comparing diagnostic test results with CRS. For the agreement analysis, the Kappa chi-square test was used. Significant *p* value of 0.05 was considered. Analysis was done by online tools such as Medcalc and GraphPad Prism 8.

## 3. Results

### 3.1. Performance of Imaging Methods

By using the USG method, out of 62 women, 12 (19.3%) were found positive for TB. Furthermore, out of 43 women, 16 (37%) were found positive by HSG (Table 1). There were findings of FGTB on laparoscopy and hysteroscopy in 19 (38%) and 15 (30%) of total women, respectively (Table 2). However, when USG, HSG, laparoscopy, and hysteroscopy were compared with CRS, 8 (12.9%), 6 (9.6%), 7 (14%), and 6 (12%) were found in the TB group, respectively.

### 3.2. Performance of Bacteriology Methods

**Figure 3:**
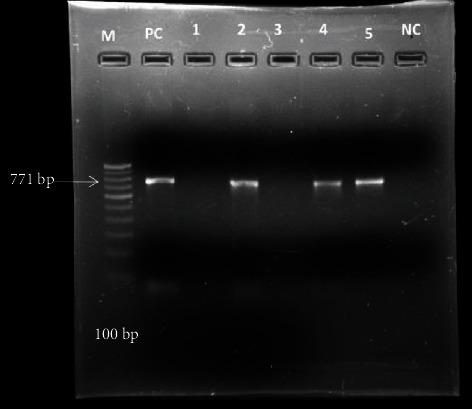
Gel image of amplified PCR product of MPT64 gene. M: marker 100 bp; PC: positive control (H37Rv); lanes 2, 4, and 5: positive band for Mycobacterium tuberculosis (*mpt64* gene); lanes 1 and 3: negative for *mpt64* gene; and NC: negative control (PCR grade water).

Out of 62 samples, 3 (4.8%) samples were found to be positive by GeneXpert and culture method. Only one sample was found to be rifampicin resistant by GeneXpert. Further, 5 isolates were also found positive by PCR (Figure 3). However, when these results were compared with CRS, similar 3 samples are found in the confirmed TB group, whereas 2 samples were found in probable TB group and 57 were in non-TB group, respectively, as shown in Table 3.

### 3.3. Performance of Histopathology Methods

Of 62 samples, 4 (6.4%) samples were found to be positive by HPE. However, when compared with CRS, 4 samples were found in confirmed TB group, and 58 were in non-TB group (Table 4).

### 3.4. Agreement of Imaging, Bacteriology, and Histopathological Results with CRS

When the kappa (*k*) value was calculated, GeneXpert and culture showed perfect agreement with CRS, each with a value of 1.0, whereas the agreement of PCR (*k* = 0.73) and HPE (*k* = 0.73) was found to be substantial with CRS. USG (*k* = 0.52) showed moderate agreement with CRS. However, HSG (*k* = 0.31), laparoscopy (*k* = 0.22), and hysteroscopy (*k* = 0.36) showed fair agreement with the CRS, respectively (Tables 1 and 2).

### 3.5. Tuberculosis in Women with Tubal Factors

A total of 43 were found to have tubal factor infertility. Out of these, only 6 (13.9%) women were characterized in the definitive TB category, whereas 10 (23.2%) samples were found in the probable TB group and 21 (48.8%) samples were categorized in the possible TB group. Among all women, tubal occlusion and tubal dilation are the most prevalent conditions for TB infection, but tubal conditions fall into probable TB and possible TB cannot be ruled out as negative TB. Three samples from the probable TB group had dual conditions (calcifications+ tubal outline irregular, tubal occlusion + peritubal adhesion, and tubal occlusion+ tubal dilation), whereas only one sample with triple conditions (calcifications + tubal outline irregular + tubal occlusion) falls into the same group of TB. One woman found definitive TB with dual tubal abnormal conditions (tubal occlusion + tubal dilation). Two women were found with normal spillage conditions. Only 6 women were categorized in non-TB group (Table 5).

## 4. Discussion

According to the guidelines for extra-pulmonary TB for India, criteria should be used to make a diagnosis of FGTB [18]. But problems arise when the presentation is variable, and a high level of clinical suspicion is necessary to make the diagnosis. Around 11% of individuals report having no symptoms other than infertility, and these patients require a diagnostic workup to rule out all prevalent causes of infertility [27].

**Figure 4:**
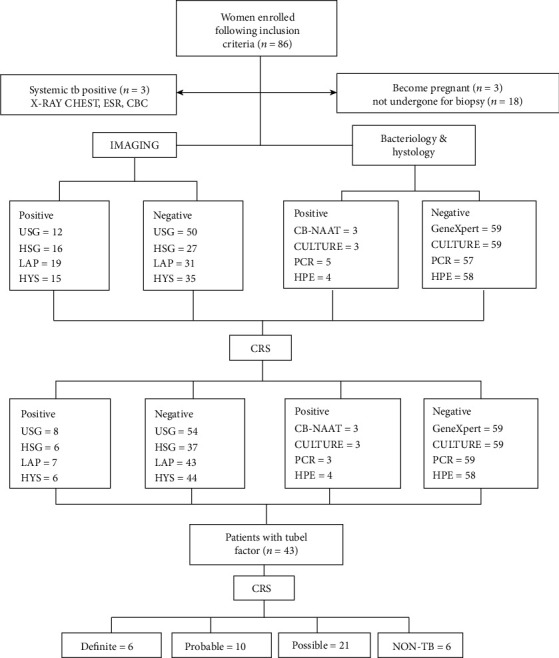
Diagnostic algorithm for FGTB. ESR: erythrocyte sedimentation rate; CBC: complete blood count; USG: ultrasonography; HSG: hysterosalpingograpgy; LAP: laparoscopy; HYS: hysteroscopy; PCR: polymerase chain reaction; HPE: histopathology; and CRS: composite reference standard.

The presence of AFB on microscopy, culture, or histopathological evidence of TB granuloma provides a definitive diagnosis, but it is only positive in a small number of cases and forces the use of additional modalities such as PCR, hysteroscopy, or laparoscopy findings to make a timely diagnosis for early treatment. However, there is no gold standard approach to detecting FGTB, so in this study, we shared the experience and reliability of our proposed criteria of CRS in the diagnosis of FGTB (Figure 4). We compared a mix of microbiological, histological, molecular, and radiological methods and history of TB. Kappa is a statistical coefficient that evaluates the degree of agreement between two raters (judges) who classify things into mutually exclusive groups [28].

In our study, the agreement between conventional investigation and CRS corresponds to the collective diagnostic model. In GeneXpert, whereas culture has perfect agreement, PCR and HPE have substantial agreement with CRS. Most of the time, endometrial biopsy does not contain sufficient numbers of bacilli for AFB or culture investigation [29]. Several investigations have been conducted in the past using endometrial tissue as well as tissues from other organs, but due to low pick-up rates (1-18%) of bacilli or most probably as a result of the monthly shedding of the endometrium's superficial layers, identification of TB was very poor [22, 30, 31].

In the imaging, USG shows moderate agreement with CRS, whereas HSG, laparoscopy, and hysteroscopy each have fair agreement, respectively. The sensitivity of the imaging and pathology tests ranged from 75.0% to 85.71%, whereas specificity was found to range from 70.27% to 98.28%. On the other hand, GeneXpert and culture have 100% sensitivity and specificity, but PCR has 100% sensitivity and 96.61% specificity. The three women who tested positive for GeneXpert also tested positive for culture and PCR which indicates active TB. Of the 5 PCR-positive samples, 3 samples were categorized as definitive TB, and 2 were in the probable TB category (Tables 5 & 6). One patient (OBG19RS23) with tubal blockage went under all diagnostic tests, and the findings in the fallopian tubes were beaded tubes and intrauterine adhesion. She was found to be GeneXpert positive with rifampicin resistance, and culture positive, PCR positive, and caseous nodules were found in HPE (Table 6). This trend indicates the presence of active bacilli in the patients. Our study is comparable to a recent study conducted by Sethi et al. [3], where PCR was found 22.39% positive and HPE 2.99%. However, no cases were found culture positive. Our findings suggest high reliability on our criteria of CRS, as clinicians can be quick to identify FGTB patients in order to avoid irreversible damage.

**Figure 5:**
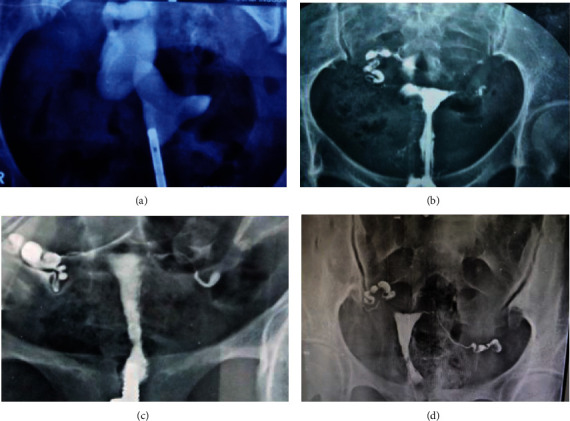
(a) HSG showing right hydrosalpinx and left cornual block; (b) HSG showing irregular uterine cavity with a localized peritoneal spill on the right and tubal occlusion on left; (c) HSG showing irregular and deformed uterine cavity with mild right hydrosalpinx; and (d) HSG showing normal uterine cavity and fallopian tubes without any peritoneal spill.

In high prevalence countries, mycobacterium culture and histopathology facilities are inadequate [32]. In that situation, the infection is often detected during HSG for the first time in any of the infertility investigations [33]. Furthermore, HSG is still the gold standard for tubal lumen assessment [20] and is a useful method for the diagnosis of female genital TB [34]. Genital TB causes a range of HSG appearances, from nonspecific to specific findings (Figure 5). According to Chavhan [34] and Afzali [14], the appearance of calcified lymph nodes in the pelvic or along the length of the fallopian tubes may confirm a diagnosis of tuberculosis. In our study, no women with calcified fallopian tubes were found to have definitive TB. But other samples which lie in the probable and possible groups cannot be ruled out as non-TB group. In tubal outline, caseous ulceration of the tubal mucosa results in an irregular, ragged, or diverticular appearance of the tubal lumen contour. Tubal occlusion (18%) is the most common finding by HSG in TB conditions in our study. Scarring can cause several constrictions along the course of the fallopian tube, giving it a “beaded” pattern. Each condition of tubal occlusion from all tubal blockage cases was also found to be prevalent (46.51%) in our study. We found HSG findings with tubal occlusion and dilation were the most prevalent conditions of FGTB in women with tubal factor infertility. The previous history is not much helpful factor to include in the model as only three women with a history of TB out of 11 women were found to be FGTB positive by CRS. The remaining 8 women with history and suffering from tubal blockage with imaging consistency were categorized in probable TB. Overall, out of 62 patients, 8 (12.9%) patients were found TB positive, in which 6 (9.6%) women with tubal blockage and 2 asymptomatic women were found to be TB positive by using CRS (Table 6). By using HSG, the typical radiographic features of genital TB are reliable indicators. All 62 samples (control) from the patients without mycobacterial infections were found to be negative for both PCR and culture as expected. The PCR assay cannot differentiate live and dead bacteria; hence, it is recommended only for new and active cases [35]. We could only include EMB samples in our study as EMB curettage is less invasive. The number of samples is less as it is a one-year study.

## 5. Conclusion

Although composite reference standard can minimize the degree of bias, they cannot entirely eliminate it since a combination of imperfect tests is unlikely to generate a composite standard with perfect sensitivity and specificity. In our study, bacteriology had a perfect and substantial agreement with CRS, so we should collect a sufficient endometrial sample using the right approach, preferably laparoscopic or hysteroscopy guided, from a highly suspicious site. The combination of imaging tests with CRS has a better scope as their agreement is fair with CRS. Due to differential causes or earlier damage of fallopian tubes by TB in infertile women, HSG manifestations have high numbers of false positivity, so high suspicion for each patient is required. Our study demonstrates that, while still using microbiological, histological, and radiological techniques, the composite reference standard incorporates many least invasive diagnostic modalities and is conducive to conclude FGTB.

## Figures and Tables

**Table 1:** Performance of the Imaging methods for the diagnosis of FGTB: sensitivity, specificity, and kappa agreement in comparison with composite reference standards.

USG	CRS		HSG	CRS	
12/62	TB group (*n* = 8)	Non-TB group (n =54)	16/43	TB group (*n* = 6)	Non-TB group (*n* = 37)
Positive (*n* = 12)	6	6	Positive (*n* = 16)	5	11
Negative (*n* = 50)	2	48	Negative (*n* = 27)	1	26
Sensitivity	80.00%, (95% CI: 44.39% to 97.48%)		Sensitivity	85.71% (95% CI: 42.13% to 99.64%)	
Specificity	88.89%, (95% CI: 77.37% to 95.81%)		Specificity	70.27% (95% CI: 53.02% to 87.13%)	
PPV	57.14%, (95% CI: 37.10% to 75.09%)		PPV	35.29%, (95% CI: 23.39% to 49.26%)	
NPV	96.00%, (95% CI: 87.81% to 98.77%)		NPV	96.30%, (95% CI: 80.71% to 99.38%)	
Kappa value (95% CI)	Agreement	Level of agreement	Kappa value (95% CI)	Agreement	Level of agreement
0.52 (0.24 to 0.81)	72.74%	Moderate	0.31 (0.053 to 0.57)	59.22%	Fair

PPV: positive predictive value; NPV: negative predictive value; TB group: TB-suspected patients; PCR: polymerase chain reaction; CRS: composite reference standard. For patients with suspicion of FGTB, diagnosis of TB was given if any two of culture/histopathology/radiological findings were positive.

**Table 2:** Performance of the Imaging endoscopic methods for the diagnosis of FGTB: sensitivity, specificity, and kappa agreement in comparison with composite reference standards.

Laparoscopy	CRS		Hysteroscopy	CRS	
19/50	TB group (*n* = 7)	Non-TB group (*n* = 43)	15/50	TB group (*n* = 6)	Non-TB group (*n* = 44)
Positive (*n* = 19)	5	14	Positive (*n* = 15)	5	10
Negative (*n* = 31)	2	29	Negative (*n* = 35)	1	34
Sensitivity	77.78% (95% CI: 39.99% to 97.19%)		Sensitivity	85.71% (95% CI: 42.13% to 99.64%)	
Specificity	67.44% (95% CI: 51.46% to 80.92%)		Specificity	77.27% (95% CI: 62.16% to 88.53%)	
PPV	33.33% (95% CI: 22.32% to 46.53%)		PPV	37.50% (95% CI: 24.34% to 52.81%)	
NPV	93.55% (95% CI: 80.76% to 98.04%)		NPV	97.14% (95% CI: 84.61% to 99.53%)	
Kappa value (95% CI)	Agreement	Level of agreement	Kappa value (95% CI)	Agreement	Level of agreement
0.22 (-0.015 to 0.468)	58.64%	Fair	0.36 (0.09 to 0.64)	65.20%	Fair

**Table 3:** Performance of the bacteriology for the diagnosis of FGTB: sensitivity, specificity, and kappa agreement in comparison with composite reference standards.

GeneXpert	CRS		Culture	CRS	
3/62	TB group (*n* = 3)	Non-TB group (*n* = 59)	3/62	TB group (*n* = 3)	Non-TB group (*n* = 59)
Positive (*n* = 3)	3	0	Positive (*n* = 3)	3	0
Negative (*n* = 59)	0	59	Negative (*n* = 59)	0	59
Sensitivity	100.00% (95% CI: 29.24% to 100.00%)		Sensitivity	100.00% (95% CI: 29.24% to 100.00%)	
Specificity	100.00% (95% CI: 93.94% to 100.00%)		Specificity	100.00% (95% CI: 93.94% to 100.00%)	
PPV	100.00%		PPV	100.00%	
NPV	100.00%		NPV	100.00%	
Kappa value (95% CI)	Agreement	Level of agreement	Kappa value (95% CI)	Agreement	Level of agreement
1.0 (1.0 to 1.0)	90.79%	Perfect	1.0 (1.0 to 1.0)	90.79%	Perfect

**Table 4:** Performance of the PCR and HPE for the diagnosis of FGTB: sensitivity, specificity, and kappa agreement in comparison with composite reference standards.

PCR	CRS		HPE	CRS	
5/62	TB group (*n* = 3)	Non-TB group (*n* = 59)	4/62	TB group (*n* = 4)	Non-TB group (*n* = 58)
Positive (*n* = 5)	3	2	Positive (*n* = 4)	3	1
Negative (*n* = 57)	0	57	Negative (*n* = 58)	1	57
Sensitivity	100.00% (95% CI: 29.24% to 100.00%)		Sensitivity	75% (95% CI: 19.41% to 99.37%)	
Specificity	96.61% (95% CI: 88.29% to 99.59%)		Specificity	98.28% (95% CI: 90.76% to 99.96%)	
PPV	60.00% (95% CI: 27.75% to 85.42%)		PPV	75.00% (95% CI: 28.29% to 95.78%)	
NPV	100.00%		NPV	99.28% (95% CI: 91.26% to 99.68%)	
Kappa value (95% CI)	Agreement	Level of agreement	Kappa value (95% CI)	Agreement	Level of agreement
0.73 (0.38 to 1.0)	87.88%	Substantial	0.73 (0.37 to 1.0)	87.93%	Substantial

**Table 5:** Findings of fallopian tube TB suspected infertility patients and clinical assessment of patients on the basis of composite reference standard (CRS) criteria.

Hysterosalpingogram *n* = 43	Clinical assessment of patients(CAP)				
	Definitive TB groups	Probable TB groups	Possible TB groups	Non-TB groups	Total
	CRS or GeneXpert or culture	PCR or history + imaging suggestive for FGTB	Imaging suggestive to FGTB	Negative for all tests	
Calcifications	—	1	2	1	4
Tubal outline irregular	1	1	2	—	4
Tubal occlusion	2	2	3	1	8
Tubal dilation	2	—	2	—	4
Peritubal adhesion	—	1	2	—	3
Calcifications+ tubal outline irregular	—	1	—	1	2
Calcifications+ tubal outline irregular+ tubal occlusion	—	1	2	—	3
Tubal occlusion+ peritubal adhesion	—	1	1	—	2
Tubal occlusion+ tubal outline irregular	—	—	1	1	2
Tubal occlusion+ calcifications+ peritubal adhesion	—	—	1	—	1
Tubal occlusion+ tubal dilation	1	1	2		4
Tubal outline irregular+ peritubal adhesion		—	1	1	2
Tubal dilation+ calcifications (hydrosalpinx)	—	—	1	1	2
Normal spills	—	1	1	—	2
Total	6	10	21	6	43

**Table 6:** Clinical findings in patients of genital tuberculosis by using CRS.

Patients no.	Methods	Findings
OBG19RS 14	USG, HSG, hysteroscopy, GeneXpert, culture, PCR	Diffused endometrial border, dilated tube, hydrosalpinx, GeneXpert positive, culture positive, PCR positive
OBG19RS 23	USG, HSG, laparoscopy, hysteroscopy, GeneXpert, culture, PCR	Beaded tubes, intrauterine adhesion, GeneXpert positive, culture positive, PCR positive with rifampicin resistant, caseous nodules
OBG19RS 38	History, USG, HSG, laparoscopy, hysteroscopy, GeneXpert, culture, PCR	Localized peritoneal spill with tubal occlusion, GeneXpert positive, culture positive, PCR positive, tubercular nodules
OBG19RS 40	History, USG, HSG, laparoscopy, hysteroscopy	Bilateral tubal dilation, pyosalpinx
OBG19RS 44	USG, HSG, laparoscopy, HPE	Tubal occlusion and dilation, cornual block
OBG19RS 49	USG, HSG, laparoscopy, hysteroscopy, HPE	Outline irregular, visualized endometrial disease-like tubercles
OBG19RS 58	History, USG, laparoscopy, hysteroscopy, HPE	Intrauterine adhesions, heterogeneous endometrium with irregular surface, epithelial granulomatous nodules
OBG19RS 59	USG, laparoscopy, HPE	Endometritis, lesions on uterus like tubercles, caseous nodules

## Data Availability

The data used to support the findings of this study are available from the corresponding author upon request.
